# Seascape connectivity of European anchovy in the Central Mediterranean Sea revealed by weighted Lagrangian backtracking and bio-energetic modelling

**DOI:** 10.1038/s41598-020-75680-8

**Published:** 2020-10-29

**Authors:** Federico Falcini, Raffaele Corrado, Marco Torri, Maria Cristina Mangano, Rafik Zarrad, Antonio Di Cintio, Luigi Palatella, Othman Jarboui, Hechmi Missaoui, Angela Cuttitta, Bernardo Patti, Rosalia Santoleri, Gianluca Sarà, Guglielmo Lacorata

**Affiliations:** 1 Istituto di Scienze Marine, Consiglio Nazionale delle Ricerche, Via Fosso del Cavaliere 100, 00133 Rome, Italy; 2 Istituto per lo studio degli impatti Antropici e Sostenibilità in ambiente marino, Consiglio Nazionale delle Ricerche, Via del mare 3, 91021 Torretta-Granitola, Campobello di Mazara (TP), Italy; 3 Dipartimento di Scienze della Terra e del Mare (DiSTeM), Università degli Studi di Palermo, Viale delle Scienze Ed. 16, 90128 Palermo, Italy; 4 Dipartimento Ecologia Marina Integrata, Stazione zoologica Anton Dohrn, Lungomare Cristoforo Colombo (complesso Roosevelt), 90142 Palermo, Italy; 5 Institut National des Sciences et Technologies de la Mer, BP 138, 5199 Mahdia, Tunisia; 6 Liceo Scientifico Statale “C. De Giorgi”, viale De Pietro 14, 73100 Lecce, Italy; 7 Institut National des Sciences et Technologies de la Mer, BP 1035, 3018 Sfax, Tunisia; 8 Institut National des Sciences et Technologies de la Mer, 2025 Salambô, Tunisia; 9 Istituto di Studi sul Mediterraneo, Consiglio Nazionale delle Ricerche, Via Filippo Parlatore 65, 90145 Palermo, Italy; 10 Istituto per lo studio degli impatti Antropici e Sostenibilità in ambiente marino, Consiglio Nazionale delle Ricerche, Lungomare Cristoforo Colombo 4521, 90149 Palermo, PA Italy; 11 Center of Excellence for Telesensing of Environment and Model Prediction of Severe events (CETEMPS), Università dell’Aquila, via Vetoio snc (Fraz. Coppito), 67100 L’Aquila, Italy

**Keywords:** Fisheries, Physical oceanography

## Abstract

Ecological connectivity is one of the most important processes that shape marine populations and ecosystems, determining their distribution, persistence, and productivity. Here we use the synergy of Lagrangian back-trajectories, otolith-derived ages of larvae, and satellite-based chlorophyll-a to identify spawning areas of European anchovy from ichthyoplanktonic data, collected in the Strait of Sicily (Central Mediterranean Sea), i.e., the crucial channel in between the European and African continents. We obtain new evidence of ecosystem connectivity between North Africa and recruitment regions off the southern European coasts. We assess this result by using bio-energetic modeling, which predicts species-specific responses to environmental changes by producing quantitative information on functional traits. Our work gives support to a collaborative and harmonized use of Geographical Sub-Areas, currently identified by the General Fisheries Commission for the Mediterranean. It also confirms the need to incorporate climate and environmental variability effects into future marine resources management plans, strategies, and directives.

## Introduction

Seascape, ecological spatial connectivity is one of the most important processes affecting distribution, persistence, and productivity of coastal marine populations. Connectivity relies on dispersion of young larvae (or algal spores) by ocean currents and it is crucial for designing and managing fishing and protecting activities^1,2^. High spatial connectivity among distant ecosystems, indeed, may guarantee replenishment of populations and nutrient availability. Recognition of spatial connectivity is at the base of the understanding of ecological processes and the evolution of marine resources, in both coastal and open ocean ecosystems (e.g., pelagic, epipelagic, etc.)^3–5^.

Mechanistic reconstruction of spawning areas of a small pelagic fish species, representing a shared stock of highly recognized economic values, is a fundamental step for expanding our knowledge on spatial scales, patterns, and rates of connectivity. Ad hoc investigations in this particular field may enable managers to design and apply specific strategies, beyond geographical borders, also promoting a more effective management of fishery resources^6,7^. In this context, dynamic oceanographic structures have a crucial role^8–12^. The effect of transport efficiency on the early life stage of fishes is, indeed, at the base of the understanding distributions of small pelagic species showing short life span, high fecundity, and a vulnerable planktonic stage among their life-history traits^13^.

From ecological perspective, small pelagic fish resources play a key role in functioning of marine systems because of their intermediate position in the trophic web. In this context, the European anchovy *Engraulis encrasicolus* represents one of the most important species in the Mediterranean Sea. Its distribution shows independent stock units and irregular spatial patterns that were justified by the strong control that environmental variability has on spawning areas and rates, recruiting, and landing^14^.

**Figure 1:**
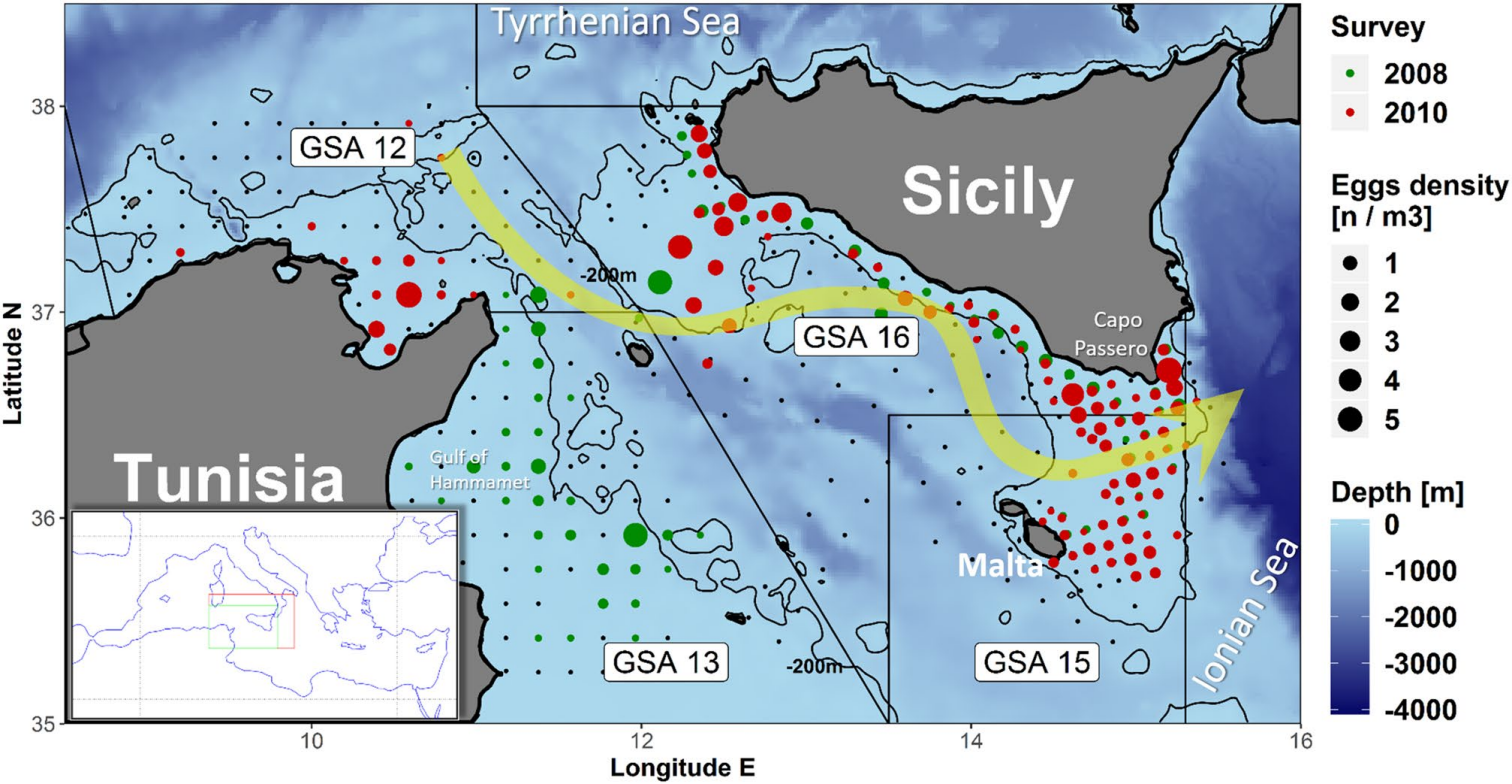
Spatial distribution of anchovy eggs in both sides of the Strait of Sicily, as obtained by the ichthyoplanktonic survey, conducted in the summers 2008 and 2010 in the two study areas^22,60^. The solid curves represent the 200-m depth isoline; the yellow arrow represents the climatologic path of the AIS^19^. The inner panel indicates the Strait of Sicily (green box) within the Mediterranean Sea; the red box indicates the broader area that results from the Lagrangian back-tracking (Figs. 3 and 4), as well as the area considered for the DEB modelling (Fig. 5).

Here we focus on European anchovy within the Strait of Sicily (i.e., the channel in between the European and African continents that connects the Eastern and Western Mediterranean sub-basins), whose reproductive strategy is strongly controlled by the surface circulation^15–18^, mainly characterized by the meandering pattern of the Atlantic Ionian Stream (AIS)^19^ (Fig. 1). In general, changes of AIS spatial pattern can enlarge or reduce the area occupied by its cyclonic meanders, and displace its anti-cyclonic part that is usually close to the coast^15^ (Fig. 1).

Previous Lagrangian simulations estimated the inter-annual variability of the efficiency of coastal currents in connecting spawning to recruitment areas of European anchovy off the southeastern coast of Sicily^23^. Such an approach also brought to mechanistic explanations of those mesoscale, wind-induced dynamics that rule dispersion of larvae in that particular area^17,18^. It results that, during the anchovy summer spawning season, the AIS and mesoscale coastal currents act as a conveyor belt that advects anchovy eggs (and larvae) from the spawning sites (located over the continental shelf off the southern coast of Sicily) toward the southernmost part of the island, i.e., off Cape Passero (Fig. 1). There, the frontal structure that originates from the meeting of AIS and Ionian Sea water masses tends to promote a major productivity of Sicilian-Maltese area, resulting in concentration processes that are favorable for growth and survival processes of anchovy larvae^15,20^.

Beyond the importance of the frontal area off Cape Passero for the anchovy reproductive ecology, it is also worth noting how the generally more favorable environmental conditions over the whole continental shelf areas of the Strait may be relevant in determining strength of annual anchovy recruitment. Recent studies evidenced a strong relationship between the estimated rate of occurrence of anchovy late larval (pre-juvenile) stages in continental shelf areas of the Strait of Sicily, resulting from surface current advection from spawning sites, and hydro-acoustic biomass estimates of the adult fraction of the population in the following reproductive season one year later^21^.

Based on discontinuous (in space and time) planktonic datasets, collected over different Geographic Sub Areas (i.e., GSA 12, 13, 15, and 16) of the Strait of Sicily in 2008 and 2010, observational data highlighted a widespread spawning activity of European anchovy over its the continental shelves^22^ (Fig. 1). In particular, density of eggs in this region evidenced a broad spatial distribution, especially in the central sector of the Strait, where the European and African shelf breaks are closer. From these inter-regional summer samplings over different GSA, Patti et al.^22^ provided an estimation of exchange rate across the European and African sides of the Strait of Sicily. These rates are significant in terms of number of Lagrangian particles that were released in the spawning areas crossing the Strait (up to 20%). However, the same authors stated that the high mortality rate that typically affects small pelagic fish during the planktonic stage could considerably decrease the actual exchange between continents.

**Figure 2:**
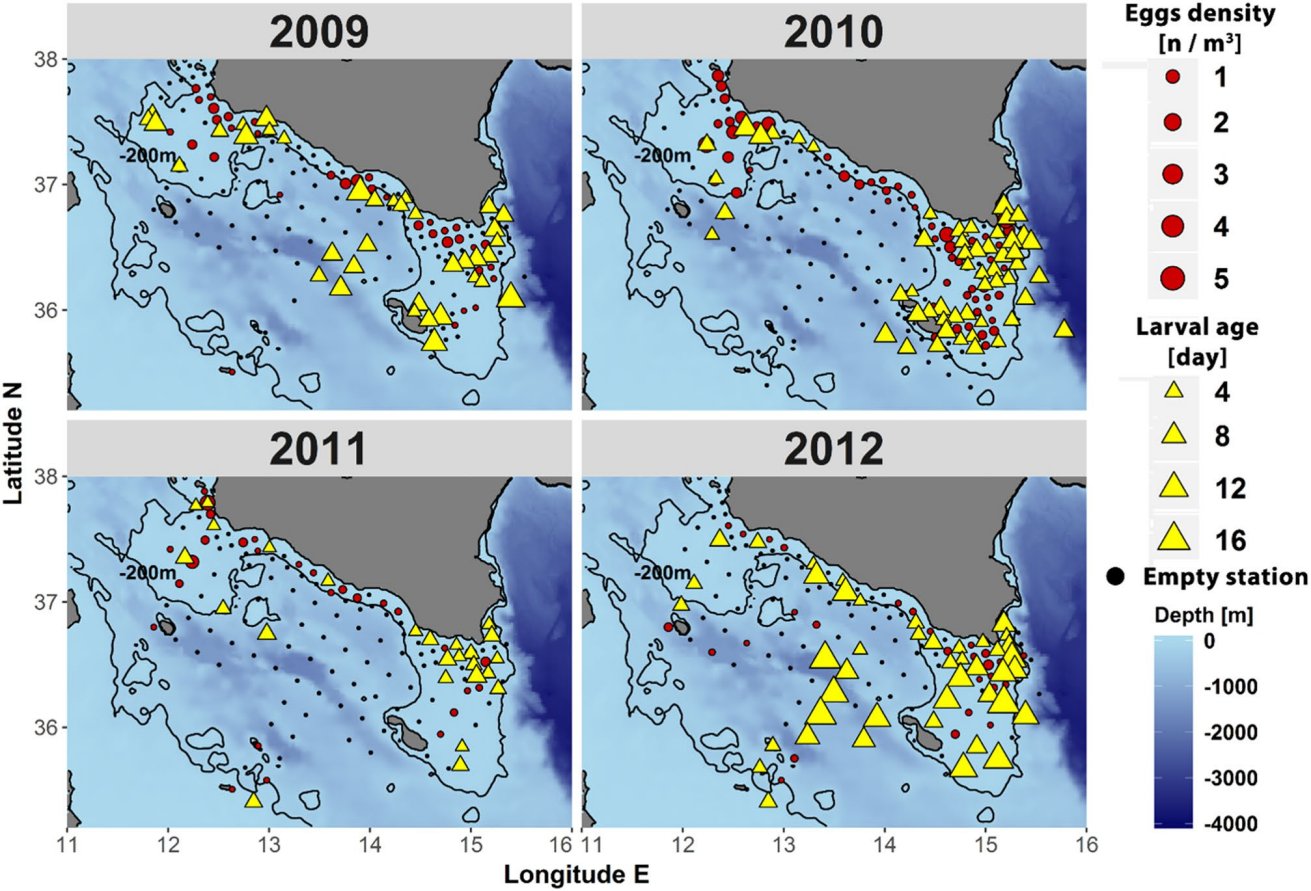
Eggs density and larval age distribution of European anchovy in the Strait of Sicily, collected in the summer period 2009–2012. Size of yellow triangles represents the mean age of anchovy larvae found in each station as estimated by the otolith analysis. The bathymetric contour of − 200 m (bold line) evidences the shape of the continental shelf in the study area. Maps were generated using R sofware version 3.4.1, R Core Team (2017). R: A language and environment for statistical computing. R Foundation for Statistical Computing, Vienna, Austria. https://www.R-project.org/.

In this framework, the effect of the environmental conditions occurring during the planktonic dispersion of anchovy larvae within the whole Strait of Sicily has never been evaluated, and it needs to be explored thoroughly. Inspired by previous studies^15,17,18,21^ and by the patchy (although stimulating) datasets^22^ we summarized in Fig. 1, here we explore Lagrangian connectivity between the European and African coastal zones in the Central Mediterranean Sea by using a novel approach, based on back-trajectory analysis. In particular, we use the continuous ichthyoplanktonic dataset that refer to the sole GSA 15 and 16 from 2009 to 2012 (Fig. 2), in order to initialize the back-in-time simulation from in situ larval ages and geographical position. Actual ages of larvae are determined from otoliths measurements, which provide reliable ending conditions for back-trajectories. This innovative approach allows recognizing the original spawning regions by going back to larva “age zero” and also accounting for egg drifting, enlightening dynamical connections among different spawning and recruitment areas. In summary, novelty of this study is, therefore, given by the fact that connectivity is not explored by tracking the evolution of Lagrangian trajectories departing from spawning areas^17, 18,21,23^; but rather from the statistical reconstruction (i.e., estimation of probability distribution functions) of spawning areas that represent arrival targets of back-in-time trajectories departing from anchovy larvae, collected in a specific region and having a specific age. Such an investigation might reveal insights on biomass dynamics for this important fish population, since recruitment strength in GSA 15 and 16 (South of Sicily) may be affected by connectivity with the surroundings GSAs and, in particular, by transport rates of eggs and early larval stages released form the continental shelf off northern Tunisia coast (i.e., GSA 12).

Lagrangian particle evolution is, however, affected by finite time predictability issues, due to nonlinear dynamics and imperfections of the model for marine currents^24^. For backward motion, therefore, a set of initially close tracer particles, confined around a given starting point, will spread its concentration over much larger scales when integrated backward-in-time. This makes the identification of potential spawning areas a non-trivial task. At this regard, here we use auxiliary information from satellite-based Chlorophyll-a field that is encountered by the larvae in their backward journey. Chlorophyll content for each trajectory provides probabilistic weight of the significance of the ending positions, thus constraining the success of each trajectory (see Methods): only those individuals that travel within Chlorophyll-rich waters have a significant survival probability.

To assess Lagrangian reconstruction of spawning areas for European Anchovy we pursue an independent, bio-energetic mechanistic approach, i.e. Dynamic Energy Budget (DEB) modelling^25^. In our application, indeed, DEB represents a synoptic opportunity to verify that the probably distribution function (pdf) of arrivals of back-trajectories correspond with coastal areas where favorable spawning environmental conditions occurs during the time range we consider for the Lagrangian analysis (i.e., 2009–2012). DEB already proved to be successful when applied to quantify the effects of environmental conditions, as well as future scenarios, on the potential distribution and vulnerability of marine resource^26–28^. By using functional trait-based mechanistic bioenergetics, these models are also able to incorporate effects of environmental drivers at levels that exceed the range of values currently operating in nature (e.g., climate projections). The obtained model outcomes are critical to parameterize population-based models and ideal to inform appropriate, proactive mitigation and adaptation strategies at scales relevant to spatial management and national and regional policy decision-making^29^.

From a multidisciplinary approach that takes into account larval age, pathways, and environmental conditions experienced during the larval transport, we provide a general view of the transcontinental advection of the planktonic stages. Our findings give quantitative support to process-based, species-oriented policies for fisheries management. These should also include best practices that take into account environmental variability in the design, application, and enforcement of current fishing management policies. In particular, we highlight that connection between neighboring ecosystems and fish stocks strengthens the need of agreed management policies for fisheries of different countries exploiting shared resources. FAO currently does so through multi-level discussion that starts from multi-country experts Working Groups and technical meetings and an advisory Committee (Scientific Advisory Committee on Fisheries), providing advice in support of the FAO General Fishery Commission for the Mediterranean. In such a context, a more thorough analysis of the level of connectivity between the Italian and Tunisian continental shelf for European Anchovy stocks could be implemented, with the goal of providing the best possible scientific information to support informed management.

## Results

### Anchovy eggs and larvae distribution

Spatial distribution of anchovy early-life stage on the northern side of the Strait of Sicily (GSA 15 and 16), estimated during the summer period 2009–2012, when the peak of the European anchovy spawning time occurs in this region^30^ (see Methods), gives insight into the main spawning/retention/concentration areas (Fig. 2). In agreement with the spawning behavior of this species, most of the eggs and larvae were found between the coast and the shelf-break (isobaths of 200 m), with higher occurrence where the continental shelf has a wider offshore extension (Adventure bank and Maltese shelf area). This pattern is particularly clear in 2010 and 2011, while surveys conducted in the 2009 and 2012 also highlighted the presence of anchovy larvae in the central offshore zone. Further information was obtained by the estimation of larval age as derived by the otolith analysis. Age ranged from 1 to 17 days after hatching (DAH) and evidenced a different distribution of the age classes among years (Supplementary Fig. S1). Larvae, found in the offshore stations, resulted to be older than the coastal ones, especially in 2012 (Fig. 2). In addition, the 2012 survey highlighted the presence of late larval stages also on the continental shelf, between Sicilian coast and the southeastern most area of the Maltese archipelago (Fig. 2).

These findings suggest a potential effect of marine currents, which could be at the root of a cross-shore transport of theses planktonic stages from the spawning areas—where most of eggs and younger larvae were concentrated—to the other zones^17,18,23^. In this context, the proximity of the European shelf with the African one raises the question on the origin of older larvae and on the connectivity issue between the both sides of the Strait of Sicily.

### Lagrangian reconstruction

The back-tracking application consists in initializing about 100 numerical trajectories from each in situ eggs and larvae sampling station of our ichthyoplanktonic dataset (Fig. 2), and tracing them back to zero age, plus two additional days for egg drifting^31^. In this way, we localize the region of origin of anchovy larvae collected in the GSA15 and 16 from 2009 to 2012, and thus, the main spawning regions of this species. However, in nonlinear dynamics, the chaotic behavior of trajectories enhances dispersion over large scales. Trajectories separate from each other exponentially in time during the early phase of the spreading^32^, they follow turbulent dispersion power-law and, finally, tend asymptotically to a standard diffusive regime if the size of the basin is large enough with respect to the spatial correlation length of the flow^33^. This means that many trajectories may travel far from the “true” pelagic tracers: a set of initially close particles, confined around a given recruitment point, will spread its concentration over much larger scales when integrated backward in time, making the identification of potential spawning areas a non-trivial task (see Methods). Moreover, growth of error between virtual and real trajectories is even more enhanced due to uncertainties in the knowledge of velocity fields (i.e., unavoidable discrepancy between model and reality)^24,34^. Therefore, to provide a reliable reconstruction of spawning areas, here we evaluate the amount of Chlorophyll encountered by each trajectory during the simulation time and use this parameter as probabilistic weight of likelihood (see Methods; Supplementary Fig. S2). Also sea surface temperature might have a potential role on assessing the likelihood of trajectories. However, during the summer spawning in the Strait of Sicily, this environmental parameter is never a limiting factor for anchovy larva^21,35^ and, therefore, we did not incorporate sea surface temperature filter in the Lagrangian modelling.

**Figure 3:**
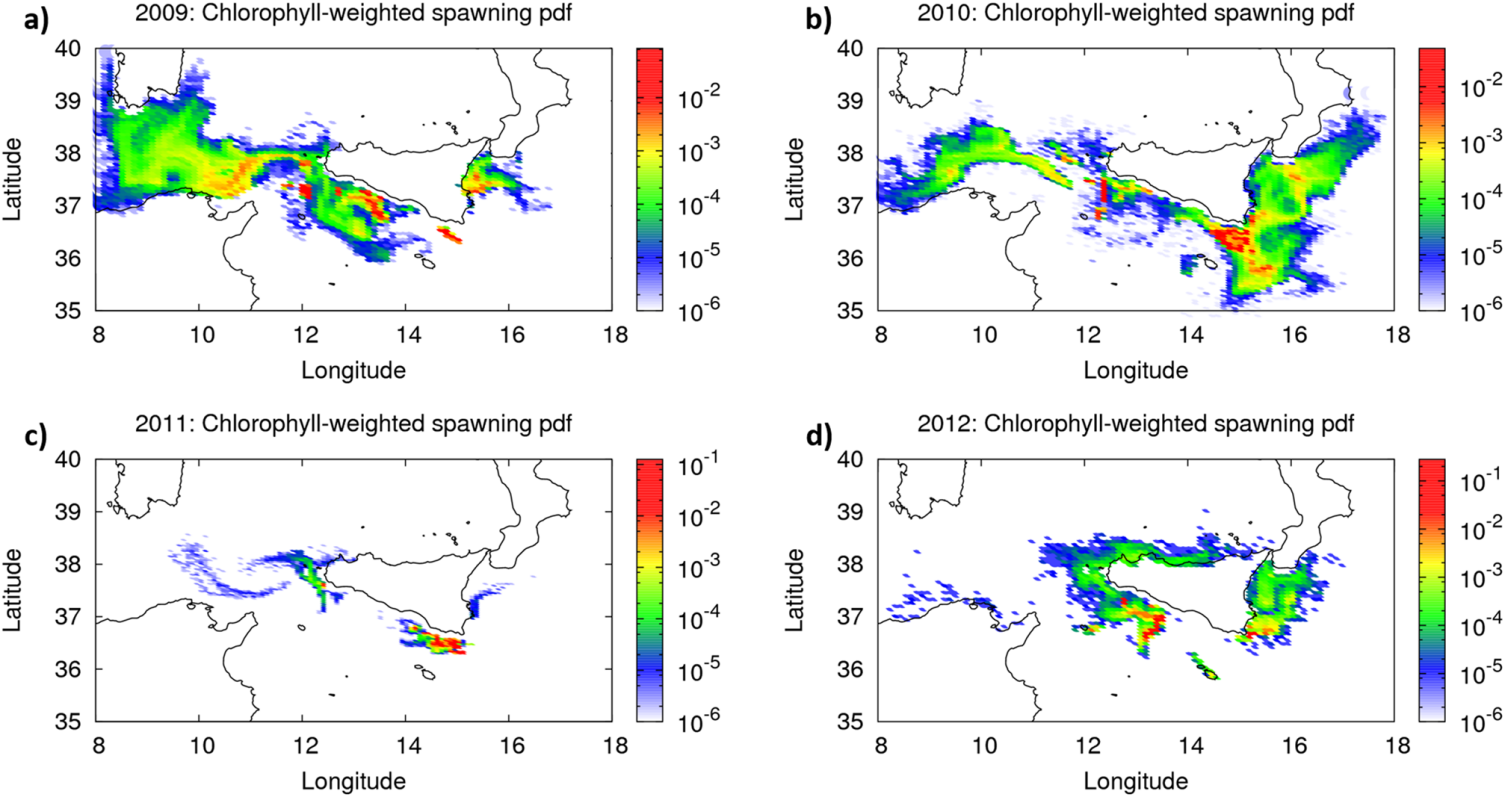
Probability distribution function (pdf) of chlorophyll-weighted spawning areas, as reconstructed from back-trajectories of anchovy larvae, collected within the GSA16, in the year 2009 (**a**), 2010 (**b**), 2011 (**c**), and 2012 (**d**). Numerical back-trajectories were initialized from in situ eggs and larvae concentration and age data (see text). Maps were generated using GIMP 2.8 (https://gimp.org).

**Figure 4:**
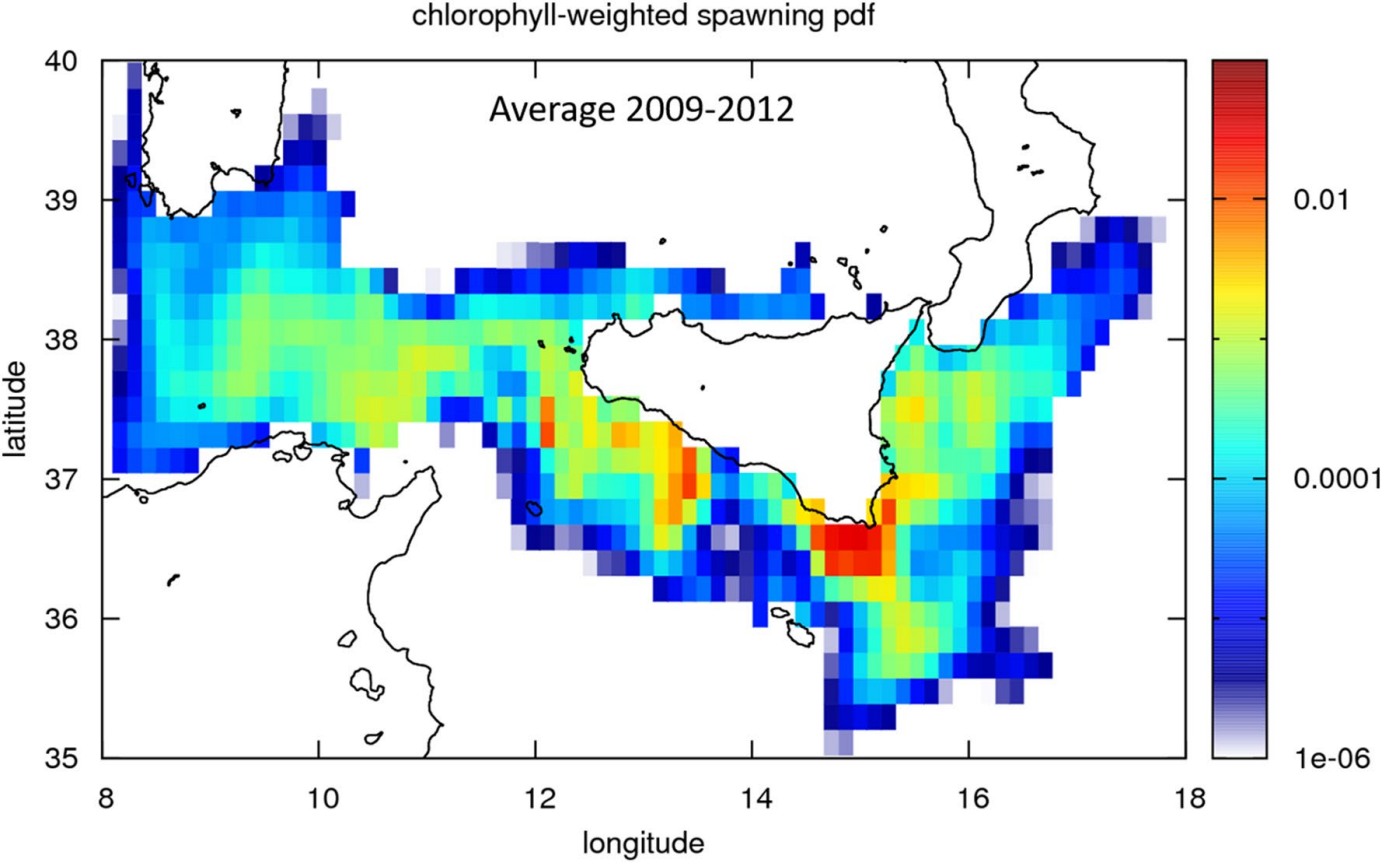
2009–2012 averaged probability distribution function (pdf) of chlorophyll-weighted spawning areas, as reconstructed from back-trajectories of anchovy larvae, collected within the GSA16. Numerical back-trajectories for each year were initialized from in situ eggs and larvae concentration and age data (see text). Map was generated using GIMP 2.8 (https://gimp.org).

Our Lagrangian, back-tracking reconstruction highlights connectivity between the three sub-basins that correspond to GSA 12, 15, and 16 (Figs. 3 and 4; Tables 1 and 2). Results confirm the main spawning areas along the southern Sicilian coast, in particular, off Sciacca and Cape Passero (Figs. 2 and 3). Moreover, chlorophyll-weighted pdf of arrivals, shows reconstructed spawning areas that are outside the GSA 15 and 16 (Figs. 3 and 4), i.e., far from the point where back-trajectories were initialized. In particular, mainly for the years 2009 and 2010, arrivals of back-trajectories in GSA12 test the hypothesis of the presence of eggs, in this region, that may potentially feed the recruiting areas of the GSA15 and 16 (Fig. 3a,b).

**Table 1:** Yearly normalized fraction of synthetic anchovy eggs that were spawned from the different GSAs, resulting from the back-trajectory analysis, without the Chlorophyll-a filter.

Year	GSA12	GSA13	GSA15	GSA16	Others
2009	0.110162	0.000654938	0.106613	0.696054	0.0865162
2010	0.0192025	0.000178107	0.234298	0.672976	0.0733451
2011	0.000594709	0.0106219	0.195358	0.770899	0.0225259
2012	0.00240981	0.054194	0.195167	0.592087	0.156142

For each year, the sum along each row represents, therefore, the total amount of larvae (normalized so that they sum to unity) that arrived in the situ sampling area (Fig. 2).

**Table 2:** Yearly normalized fraction of synthetic anchovy eggs that were spawned from the different GSAs, resulting from the back-trajectory analysis, with the Chlorophyll-a filter.

Year	GSA12	GSA13	GSA15	GSA16	Others
2009	0.187656	0.000108168	0.0702215	0.662433	0.0795809
2010	0.0388796	7.52301e−05	0.357799	0.45908	0.144166
2011	0.000688395	0	0.209173	0.78715	0.00298857
2012	0.000405269	0.00059483	0.00490244	0.872661	0.121437

For each year, the sum along each row represents, therefore, the total amount of larvae (normalized so that they sum to unity) that “survived” and thus arrived in the situ sampling area (Fig. 2).

This result is also highlighted by the normalized fraction of synthetic anchovy eggs that were spawned from the different GSAs (Tables 1 and 2); that is, the fraction of backward arrivals of synthetic larvae that were released from GSAs 15 and 16. In particular, in 2009, without the chlorophyll-a filtering process (Table 1), and thus assuming that all larvae completed the backward journey towards the spawning areas, 11% of larvae collected in the GSA 15 and 16 comes from the GSA 12 while 8% from other sectors (i.e., likely the GSA 11.2, south of Sardinia, and GSA 19, east of Sicily; see Fig. 3). When we apply the chlorophyll-a filter (Table 2), not all larvae complete the backward simulation. For this case, the percentage is then calculated in relation to the survived larvae that end up in the GSA15 and 16 (i.e., where larvae were collected). It results that, in 2009, 18% of larvae collected in these GSAs comes from the GSA 12 while 8% from other sectors. For the year 2010 we found similar results, although the percentages are lower than the 2009 case (Tables 1 and 2).

This analysis reinforces the hypothesis of a favorable, chlorophyll-rich pathway between the GSA 12 and the GSAs 15 and 16, occurring in both 2009 and 2010. Indeed, only the GSA12 increases the relative fraction of spawned larvae when applying the chlorophyll-a filter (Tables 1 and 2). The strong connectivity between GSA12 and GSA16 is also supported by the fact that the pdf of arrivals runs along the path of the AIS, which bridges the two sub areas (Fig. 1). Indeed, the meandering structure of the AIS, evident in the 2009 and 2010 (Fig. 3a,b), likely works as a conveyor belt that strongly connects the two continents, delivering eggs and early-life larvae to the Sicilian south-west coast.

### Bioenergetics modelling

The DEB theory^25^ represents a suitable tool for modelling metabolism at the whole organism level, encompassing all life-stages. Indeed, DEB model^25,36,37^ simulates the rate at which an organism assimilates and utilizes energy for its maintenance, growth and reproduction, based on those parameters that characterize species physiology and its response to environmental forcing variables (e.g., food availability and temperature), also taking into account metabolic trade-offs. DEB theory (see Methods) assumes storage of assimilated energy, which is then distributed among physiological processes^38^. This mechanism permits the exploration of time history effects, related to energetic status (feeding history) and vulnerability to factors such as temperature^39^.

Here we perform simulations to investigate potential variations in the ultimate fecundity of European Anchovy under temperature and food availability conditions observed during the study period (2009–2012). The model output, expressed in terms of Total Reproductive Output (TRO, i.e., the total number of eggs per life span), is presented in a scenario-based quantitative map^28,40^.

**Figure 5:**
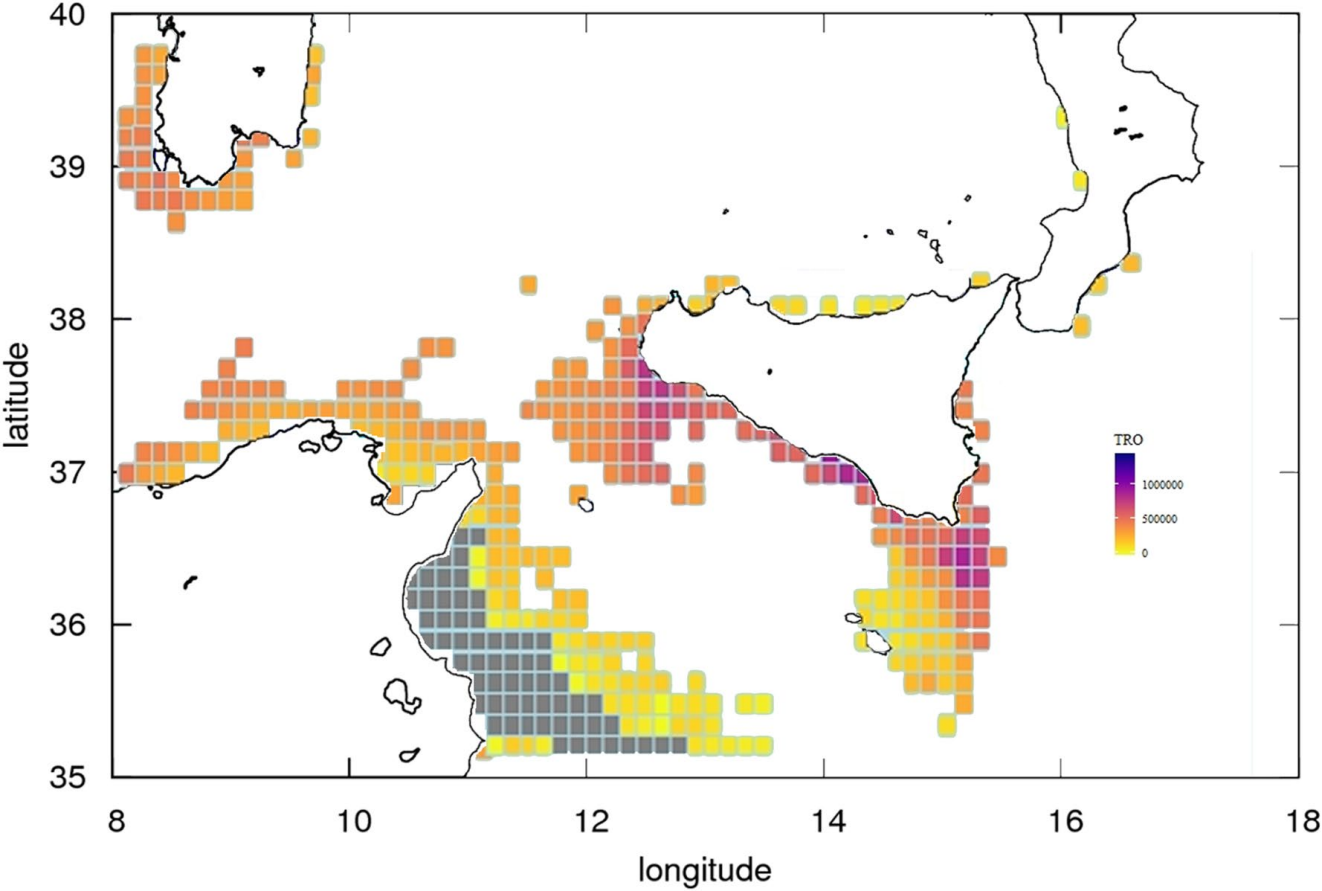
Quantitative map of Total Reproductive Output (TRO, n. eggs/n. of reproductive events, DEB model outcome) described by a continuous scale ranging (from 0 to 1,400,000) across the study area (years 2009–2012). Grey cells refer to pixel where, due to local conditions, the species do not produce eggs. Distributional range of the species was modeled within the bathymetric contour of − 200 m. Map was generated using GIMP 2.8 (https://gimp.org).

TRO simulated response shows a clear spatial heterogeneity (Fig. 5). The DEB model identifies productive areas (i.e. areas with the highest TRO values) and less productive patches (i.e. areas with the lowest TRO values) that strongly agree with the pdf of arrivals of Lagrangian back-trajectories for the years 2009–2012 (Figs. 3 and 4). Three highly productive patches (i.e., potential spawning areas; violet pixels in Fig. 5) are evident across the continental shelf off the GSA 12, 15, and 16. These three areas are also evident from both inter-annual Lagrangian results (Figs. 3) and the cumulative 2009–2012 averaged pdf of arrivals (Fig. 4); the latter plot is indeed designed to facilitate comparison between DEB and Lagrangian modelling.

In particular, low to middle range values of TRO north off Tunisia (orange pixels) indicate the potential of this area to deliver eggs and early-life larvae towards the Sicilian south-east coast. On the other hand, DEB shows zero to low egg production in the inshore part of the Gulf of Hammamet, assessing the lack of eggs that, from this region, may potentially feed the GSA 15 and 16, as it results from the Lagrangian analysis (Figs. 3 and 4).

## Discussions and conclusions

Synergy of in situ ichthyoplanktonic data and satellite-based Chlorophyll-weighted Lagrangian back tracking highlights a possible ecosystemic connectivity between GSA 12 and 16 for European Anchovy. In particular, the use of a Lagrangian modelling that statistically accounts for mesoscale and sub-mesoscale dynamics and deterministic chaos showed evidence on the role of oceanographic structures in connecting shelf areas from different GSAs, also quantifying spatial and temporal scales, as well as inter-annual variabilities and preferential pathways. Time scale of anchovy larval stage results to be comparable with the time scale of those oceanographic structures (e.g., jet, streams, and filaments) that allow for connecting the northern African shelves to the southern European ones. Indeed, besides the ability to reconstruct spawning areas, Lagrangian back-tracking highlighted the main role of the meandering structure of the AIS in connecting the ecosystems of African to European shelves in the Strait of Sicily. The analysis also shows that environmental conditions can be particularly favorable in specific years (e.g., the 2009 and 2010 in our case study), thus highlighting the crucial role of inter-annual variability and, eventually, global changes in such an ecosystem dynamics. These results are further refined by the use of biology-based, Chlorophyll-weighted back trajectories, which provided more conservative pdf of reconstructed spawning areas, and thus more robust statistical results.

Back-trajectories departs from GSAs 15 and 16 (Fig. 2), where continuous otolith growth dataset is available, and they end up to a much broader area, reaching the Tyrrhenian Sea, the area North of Tunisia, and the central sector of the Ionian Sea. In seeking to assess the zones reconstructed by Lagrangian back-tracking, DEB gives us confirmation that those areas are actually potential spawning areas for the study years (i.e., from 2009 to 2012). DEB modeling is capable to provide highly reliable, quantitative, spatially-explicit predictions of how the current (2009–2012) environmental conditions can potentially affect the European Anchovy reproduction (TRO). Interestingly, the mechanistic approach, applied by using DEB theory, assessed the reconstructed spawning areas obtained from the Lagrangian back-tracking analysis. We remark, indeed, that TROs do not depend on specific, in situ measurements and that the Lagragian back-trajectories are initialized by anchovy larvae that were collected over the sole GSA 15 and 16; nevertheless, the two approaches highlighted potential spawning areas north of Tunisia. Moreover, in absence of chlorophyll-a weighting (Supplementary Fig. S2) we obtain patterns that are similar to the filtered case: the chlorophyll-a weighting simply narrower the arrival areas and remove some off shore areas. In the light of this we notice that the DEB provides a fully independent assessment of spawning areas we reconstruct by back-trajectories.

Existing literature does not offer clear evidence of connectivity of stocks of European Anchovy among different GSAs in the Strait of Sicily. Using forward Lagrangian simulations from observed egg concentration points Patti et al.^21^ highlighted the existence of a limited exchange rate of reproductive material between both sides of the Strait. However, this exchange was thought to be negligible (on average, < 1% of total larval abundance) in the light of expected mortality rates of anchovy early stages. By using the novel Lagrangian approach (i.e., chlorophyll-a weighted back-tracking from aged larval concentration points) on a longer time series (2009–2012) and by assessing arrivals of back-trajectories with the DEB modelling and considering a longer time series, our findings confirms the time-variable exchange rate between African and European shelves, also identifying the Gulf of Tunis and the eastern zone as a key spawning area.

In particular, we notice from the Lagrangian results that the GSA12 shows reconstructed spawned eggs that can potentially feed that GSAs 15 and 16. Moreover, DEB confirms that these three GSAs are characterized by favorable spawning conditions for the whole investigated period. The synergy of these two independent results, therefore, assess the role of AIS in connecting GSA 12, 15, and 16 dynamically, thus in influencing the productivity of this cross-border area (Figs. 3 and 4).

On the other hand, zero to low egg production in the coastal area of the Gulf of Hammamet, paired with absence of back-trajectories arrivals in this region, indicates that in this Gulf favorable zone for spawning conditions during the years 2009–2012 is only limited on the edge of the continental shelf and eventual larvae that were spawned here do not statistically reach the GSA 15 and 16 in the years 2009–2012.

Our findings highlight the existence of suitable spawning and recruitment conditions occurring locally, during the advection and in the recruitments areas. In this framework, our findings suggest that mortality of anchovy larvae caused by starvation is expected to be low along their trajectories and hence indicates significant connectivity among GSAs. These results are consistent with Cuttitta et al.^41^, which characterized the genetic structure of the European anchovy populations in the Strait of Sicily. These authors found the existence of two different phylogroups probably differentiated during the Pliocene–Pleistocene period and currently not geographically separated. In agreement with our investigation, these authors also argue the presence of environmental dynamics that keep blended these two phylogroups in the central Mediterranean Sea.

Seascape connectivity is definitely affected by environmental dynamics. Inter-annual variability characterizes the hydrodynamic processes, ruling the fate and distribution of small pelagic fish larvae and juveniles amongst GSAs. This, in turn, affects biomass productivity. Moreover, inter-annual variability suggests the need of more dynamic fisheries management policies, able to integrate environmental changes and to adapt to resources changing conditions^42^. Therefore, diagnosis of environmental processes, and their changes, turn to be useful for connecting the dots between causes and effects in biomass productivity, and thus it can work as a predictive tool when it is used along with future scenarios, such those provided by the Intergovernmental Panel on Climate Change (IPCC).

Linkage networks and connectivity are both crucial knowledge to integrate when designing effective fishery management strategies. Our work sheds some light over the existence of some level of connectivity between GSA 16 and GSA 12, and provide species-specific evidence that moves toward a more ecologically sound reframing of marine resources management when dealing with cross-border areas. In any event, the current GSA framework does not prevent the implementation of regional scientific and/or management strategies involving more GSAs simultaneously. Surely, a deeper analysis about the level of connectivity existing between the Italian and Tunisian European Anchovy stocks could clarify the extent to which fishing in one GSA has the potential of affecting current and future fishing in the neighboring GSA. If deemed necessary by further investigations, cooperation among interested countries could be encouraged at the regional level, with the ultimate goal of promoting a more informed management of Mediterranean fisheries.

Our analysis was based on European Anchovy in the Strait of Sicily, which is strongly characterized by its own spawning strategy. Results of this analysis largely depend on how this strategy interacts with the sea surface dynamics of marine currents. All this, therefore, suggests that the management of biological resources needs to integrate also species-oriented evidence, since different pelagic species do not necessarily rely on the same environmental conditions and dynamics.

## Methods

### Ichthyoplanktonic dataset

Spatial distribution of anchovy early-life stage was estimated from ichthyoplanktonic surveys in the GSA 16 from 2009 to 2012. During each survey, the same systematic sampling was carried out following a regular grid of stations (1/10° × 1/10° on the continental shelf and 1/5° × 1/5° further offshore). Mesozooplanktonic samples were collected in the study area using a bongo net (40-cm opening) towed obliquely from the surface to a 100-m depth, equipped with a 200-µm mesh size net. In each tow, the volume of filtered water was estimated using mechanical flowmeters (General Oceanics Inc., FL, USA). Over the period included in the analyses for anchovy eggs data, the average number of sampling stations was 154.

Samples were immediately fixed after collection and preserved in a 70% alcohol solution for further analysis in laboratory by stereomicroscopy. Anchovy larvae were identified in a land-based laboratory and otoliths were extracted for the age determination. Both *sagittae* were extracted using insect pins and fixed on a slide using a mounting medium for microscopy. Daily rings were counted at 1000× magnification (oil immersion) starting from the spawning mark^31^; each otolith was read blind (i.e., with no size or collection location information available) by a single reader^43^. When all otoliths were completed, the process was repeated. If the difference in age between the 2 readings was < 5%, one was chosen randomly as the final read. If there was a discrepancy in age of ≥ 5% between the 2 readings, the otolith was read a third time. If after a third read a high discrepancy in the readings remained, the otolith was discarded.

No use of live vertebrates has been required for this study and no specific permissions were needed for the sampling activities since *Engraulis encrasicolus* is commercially harvested (neither endangered nor protected) and it was caught in areas where fishing is allowed. Sampling and analysis protocols are in accordance with the European Union regulations (EU Dir 2010/63).

### Lagrangian modeling

Lagrangian approaches use numerical particle trajectories, as obtained from velocity field of marine currents. What matters most to the problem we are dealing with is the role of the spatial and temporal resolution of the velocity fields. In our case the Mediterranean Forecasting System (hereafter MFS)^44–46^. The MFS model output consists in daily fields defined on a regular horizontal spatial grid, with 1/16 × 1/16 degree step, extended over 72 unevenly spaced vertical levels in depth. Considering that the Rossby first radius of deformation is *O*(10) km for the Mediterranean Sea, the MFS spatial resolution is not accurate enough to reconstruct mesoscale and sub-mesoscale structures in the study area.

MFS spatial resolution (~ 6.5 km × 6.5 km) does not allow an accurate reconstruction of mesoscale and sub-mesoscale structures (see Methods). Numerical experiments on predictability issues, related to Lagrangian simulations with the MFS model, demonstrate that the relative dispersion between tracer trajectories in the mesoscale and sub-mesoscale range is, indeed, significantly underestimated when compared to Mediterranean drifter data^24^. To compensate this drawback, we hire a kinematic modelling technique (see Methods), which has been recently set up and applied to numerical studies of transport and dispersion in the Mediterranean Sea^17,23,24,47^.

Therefore, to the MFS Eulerian fields for horizontal marine currents, we add two different versions of the Kinematic Lagrangian Model (hereafter KLM). The two-dimensional version, called KLM2, is a superposition of N_m_ independent modes, each associated to a given wavenumber k_n_, which form the following velocity components u_klm2_ (zonal) and v_klm2_ (meridional):

1 $${\text{u}}_{\text{klm2}}(\mathrm{x},\mathrm{y},\mathrm{t})=\sum_{\text{n} = \text{1}}^{{\text{N}}_{\text{m}}}{\text{A}}_{\text{n}}\text{ sin}[{\text{k}}_{\text{n}}{\text{x}}-{\text{k}}_{\text{n}}{\updelta}_{\text{n}}\sin\left({\upomega}_{\text{n}}{\text{t}}\right)]\text{cos} [{\text{k}}_{\text{n}}{\text{y}}-{\text{k}}_{\text{n}}{\updelta}_{\text{n}}\sin\left({\upomega}_{\text{n}}\text{t}+\upvarphi\right)]$$

2 $${\text{v}}_{\text{klm2}}(\mathrm{x},\mathrm{y},\mathrm{t})=-\sum_{\text{n} = \text{1}}^{{\text{N}}_{\text{m}}}{\text{A}}_{\text{n}}\text{ cos [}{\text{k}}_{\text{n}}{\text{x}}-{\text{k}}_{\text{n}}{\updelta}_{\text{n}}\sin\left({\upomega}_{\text{n}}{\text{t}}\right)]\text{sin} [{\text{k}}_{\text{n}}{\text{y}}-{\text{k}}_{\text{n}}{\updelta}_{\text{n}}\sin\left({\upomega}_{\text{n}}\text{t}+\upvarphi\right)]$$

where: x and y are zonal and meridional coordinates, respectively, of a Lagrangian particle; A_n_ is the velocity scale associated to the mode k_n_; δ_n_ and ω_n_ are amplitude and pulsation, respectively, of the periodic oscillations of the velocity modes, and φ is phase difference (common to all modes) between x and y assigned to these oscillations^24^. Here, for the KLM2, the range [k_1_, k_Nm_] corresponds to the inertial range of mesoscale turbulence of the Mediterranean Sea, as observed from experimental data. The velocity scales of the modes refer to the corresponding spatial scales via a Kolmogorov ‘− 5/3’ spectrum (two-dimensional inverse cascade): A_n_ ∝ (εk_n_)^−1/3^ where ε indicates the mesoscale 2D turbulent dissipation rate, of the order of 10^–9^ m^2^ s^−3^. This value is obtained from the horizontal dispersion that was estimated from experimental Lagrangian buoys^24^, released in the Mediterranean Sea (https://marine.copernicus.eu/, product: INSITU_MED_NRT_OBSERVATIONS_013_035).

The oscillation parameters, δ_n_ and ω_n_, control the ‘level of chaos’ of the Lagrangian trajectories: if they are both (or even only one of them) set to zero, the trajectories are regular and confined to periodic orbits; if they are assigned suitable values, Lagrangian Chaos can play its role at full regime^48^.

For the mixed layer Lagrangian modelling, we use a three-dimensional version, called KLM3, which has three velocity components, i.e., u_klm3_ (zonal), v_klm3_ (meridional), and w_klm3_ (vertical), defined as superposition of N′_m_ modes:

3 $${\text{u}}_{{{\text{klm3}}}} \left( {{\text{x}},{\text{y}},{\text{z}},{\text{t}}} \right) = \mathop \sum \limits_{{\text{n } = \text{ 1}}}^{{N_{m}^{{\prime}} }} {\text{A}}_{{\text{n}}} {\text{ sin [k}}_{{\text{n}}} {\text{x}} - {\text{k}}_{{\text{n}}} {\updelta }_{{\text{n}}} {\sin}\left( {{\upomega }_{{\text{n}}} {\text{t}}} \right){]\text{cos[k}}_{{\text{n}}} {\text{z}} - {\text{k}}_{{\text{n}}} {\updelta }_{{\text{n}}} {\sin}\left( {{\upomega }_{{\text{n}}} {\text{t} + \upvarphi ^{\prime}}} \right)]$$

4 $${\text{v}}_{{{\text{klm3}}}} \left( {{\text{x}},{\text{y}},{\text{z}},{\text{t}}} \right) = - \mathop \sum \limits_{{\text{n } = \text{ 1}}}^{{N_{m}^{{\prime}} }} {\text{A}}_{{\text{n}}} {\text{ sin [k}}_{{\text{n}}} {\text{y}} - {\text{k}}_{{\text{n}}} {\updelta }_{{\text{n}}} {\sin}\left( {{\upomega }_{{\text{n}}} {\text{t} + \upvarphi }} \right){\text{]cos [k}}_{{\text{n}}} {\text{z}} - {\text{k}}_{{\text{n}}} {\updelta }_{{\text{n}}} {\sin}\left( {{\upomega }_{{\text{n}}} {\text{t} + \upvarphi ^{\prime}}} \right)]$$

5 $$\begin{aligned}{\text{w}}_{{{\text{klm3}}}} \left( {{\text{x}},{\text{y}},{\text{z}},{\text{t}}} \right) &= - \mathop \sum \limits_{{\text{n } = \text{ 1}}}^{{N_{m}^{^{\prime}} }} {\text{A}}_{{\text{n}}} {\text{ cos [k}}_{{\text{n}}} {\text{x}} - {\text{k}}_{{\text{n}}} {\updelta }_{{\text{n}}} {\sin}\left( {{\upomega }_{{\text{n}}} {\text{t}}} \right){\text{]sin [k}}_{{\text{n}}} {\text{z}} - {\text{k}}_{{\text{n}}} {\updelta }_{{\text{n}}} {\sin}\left( {{\upomega }_{{\text{n}}} {\text{t} + \upvarphi ^{\prime}}} \right)] \\ &\quad+\sum_{\text{n} = \text{1}}^{{N}_{m}^{^{\prime}}}{\text{A}}_{\text{n}}\text{ cos [}{\text{k}}_{\text{n}}{\text{y}}-{\text{k}}_{\text{n}}{\updelta}_{\text{n}}\sin\left({\upomega}_{\text{n}}\text{t}+\upvarphi\right)]\text{sin [}{\text{k}}_{\text{n}}{\text{z}}-{\text{k}}_{\text{n}}{\updelta}_{\text{n}}\sin\left({\upomega}_{\text{n}}\text{t}+\upvarphi^{\prime}\right)]\end{aligned}$$

Here, *x*, *y*, and *z* are zonal, meridional, and vertical coordinates, respectively, of a Lagrangian particle; φ and φ’ are phase differences of the periodic oscillations between the three directions; [k_1_,k_N’m_] is the inertial range of the model; A_n_ and k_n_ are related by some scaling property, e.g., a Kolmogorov-type of spectrum, in this case, describing a 3D direct cascade^23,24^. We observe that both KLM2 and KLM3 are conservative dynamical systems, i.e*.*, the divergence of the velocity field is everywhere null. The number of modes in KLM3 does not have to be large since, in actual facts, what concerns most to our study is a good efficiency of vertical mixing, and this is accomplished just with a few velocity components.

By indicating with U_mfs_, V_mfs_, and W_mfs_ the three components of the ocean model velocity field, the full equations, which determine the evolution of the Lagrangian coordinates can be written as:

6 $$\frac{\mathrm{dx}(\mathrm{t})}{\mathrm{dt}}= {\mathrm{U}}_{\mathrm{mfs}}\left(\mathrm{x},\mathrm{y},\mathrm{z},\mathrm{t}\right)+{\mathrm{u}}_{\mathrm{klm}2}\left(\mathrm{x},\mathrm{y},\mathrm{z},\mathrm{t}\right)+{\mathrm{u}}_{\mathrm{kml}3}(\mathrm{x},\mathrm{y},\mathrm{z},\mathrm{t})$$

7 $$\frac{\mathrm{dy}(\mathrm{t})}{\mathrm{dt}}= {\mathrm{V}}_{\mathrm{mfs}}\left(\mathrm{x},\mathrm{y},\mathrm{z},\mathrm{t}\right)+{\mathrm{v}}_{\mathrm{klm}2}\left(\mathrm{x},\mathrm{y},\mathrm{z},\mathrm{t}\right)+{\mathrm{v}}_{\mathrm{kml}3}(\mathrm{x},\mathrm{y},\mathrm{z},\mathrm{t})$$

8 $$\frac{{{\text{dz}}\left( {\text{t}} \right)}}{{{\text{dt}}}} = {\text{ W}}_{{{\text{mfs}}}} \left( {{\text{x}},{\text{y}},{\text{z}},{\text{t}}} \right) + {\text{w}}_{{{\text{klm}}2}} \left( {{\text{x}},{\text{y}},{\text{z}},{\text{t}}} \right) + {\text{w}}_{{{\text{kml}}3}} \left( {{\text{x}},{\text{y}},{\text{z}},{\text{t}}} \right).$$

To mimic daily vertical migration (DVM) we also use the additional term^23^.

9 $$\frac{{{\text{dz}}\left( {\text{t}} \right)}}{{{\text{dt}}}} = { } - \gamma \left[ {{\text{z}}\left( {\text{t}} \right) - {\text{z}}_{0} } \right],$$

where transfer rate γ is ≃ 0.1 h^−1^ and z_0_ is set to 3 (day time) and 100 m (night time), depending on daylight.

Otolith georeferenced data, which ranged between 1 and 17 days post hatching, are used to generate a large number (i.e., 100 individuals) of virtual larvae/eggs with a given age distribution. The backtracking during the larval period assumes that the growth rate is a normal variable with 0.49 mm/day (mean), and 0.05 mm/day standard deviation. The temporal extension of backward simulation is defined according to the planktonic stage duration, i.e. the sum of the egg and larval period^31^. Regarding the egg stage, we considered representative a period of 48 h, which is the incubation time of this species at the temperature of ~ 22 °C^49^.

### Biological constraint for Lagrangian trajectories

In nonlinear dynamics, the chaotic behavior of trajectories enhances dispersion over large scales. The same occurs for backward motion if the system is with good approximation conservative (*i.e.*, Hamiltonian-like), as the ocean is. Hence, the identification of the origin of a tracer is largely affected by this problem and, consequently, concentration of traced particles may spread over unreliable large areas when integrated backward-in-time.

We therefore evaluate the amount of Chlorophyll encountered by each *i* trajectory during the simulation time by integrating the Chlorophyll concentration, i.e., ${\rho }_{i}^{Chl}\left(\overrightarrow{x}\right)$, along trajectories $\overrightarrow{{x}_{i}}\left(t\right)$:

10 $$\overline{{\rho_{{}}^{Chl} }}_{i} = \mathop \int \nolimits_{T}^{0} \rho^{Chl} \left( {\overrightarrow {{x_{i} }} \left( t \right)} \right)dt.$$

We then calculate the pdf of “zero-age positions” that satisfies the condition ${\overline{{\rho }^{Chl}}}_{i}>{\rho }_{min}^{Chl}$, where ${\rho }_{min}^{Chl}=\frac{1}{2}K$ and $K$ = 0.09 mg m^−3^, i.e., half of the saturation value for anchovy larvae feeding^50^.

This idea is justified by the relationship between the pelagic growth rate and the Chlorophyll concentration: only those individuals that travel within Chlorophyll-rich marine waters have a significant survival probability. In this way, we have a criterion to rule out all the final positions of the simulated backward trajectories, which have poor or null Chlorophyll content.

### Bioenergetic modeling

DEB represents a reliable and powerful tool to mechanistically describe the whole life cycle of an organism individual performance and to make predictions of life-history traits^27, 40,50–52^. In particular, DEB is a complex mechanistic model that relies on several differential equations that are solved to obtain the final amount of energy allocated to vital functions, such as metabolic maintenance and to growth and reproduction^53^. Here we adapted this model to the European anchovy and we adopted the standard version of the DEB model, which considers one reserve, one structure compartment and isomorphic growth^54^.

The energy gathered through feeding processes is stored in a reserve pool, from which it is allocated according to the κ-rule: part of the energy (κ) sustains the somatic tissues and the growth of structures, while the rest (1 − κ) maintains the maturity level and maturation or gamete production in adults. Temperature controls the rates of all energetic flows and it follows the Arrhenius rules within the thermal-tolerance range^26,54,55^. The Type II functional response^56^ instead models the relationship between food density and ingestion rate^54^. DEB theory therefore allows, through the explicit modeling of energy and mass fluxes through organisms, to derivate individual performance in terms of the most important life-history traits of a species such as, for instance, the total reproductive output (TRO) and maximum length.

In the present study, we followed a well-tested spatially explicit contextualized approach already successfully adopted in several companion studies^27,40,57^. This approach consists in running DEB models in each spatial pixel of the study area using organismal body temperature (which is assumed to be similar to the Sea Surface Temperature, as extracted through satellite imagery for every single pixel) and environmental food availability. Food availability was expressed as density (wet mass mg m^−3^), which for anchovy primarily comprise zooplankton, and obtained as a spatially continuous dataset on the distribution of food throughout the study area^40^. Nevertheless, to run DEB model in each pixel with local temperature and food density is a long computational process. Thus, due to the large number of pixels of the study area, we moved the standard DEB model from the original Kooijman’s Matlab code (https://www.bio.vu.nl/thb/)—once adapted it using anchovy DEB parameters^40^—to an R code. In doing so, we were able to automatize and speed up the process since R coding improve the computational effort and allows running DEB models at larger spatial and temporal scales^27,40,58^. The coding re-arrangement was performed by the Ecology Lab of the University of Palermo, which is one among the DEB node of the DEB world net (https://www.debtheory.org/wiki/index.php?title=DEBnet).

Our simulations were restricted to the continental shelf, based on depth (from 0 to 200 m below sea level). A vector polygon grid feature class of 346 square cells (having a size of 0.11° × 0.11° [~ 150 km^2^]) covering the study area was used. Food availability is an important forcing of the model and, for anchovy, primarily comprises zooplankton^52^. Since locally collected data for zooplankton were spatially and temporally fragmented due to sampling effort, we followed the recent approach proposed by Strömberg et al.^59^ and applied by Mangano et al.^28,40^ to obtain a continuous (space and time wise) dataset on the distribution of food throughout the study area and across time (i.e. weekly Net Primary Productivity was transformed into wet mass of zooplankton). Due to the short life span of the anchovy (~ 4 years), we extracted daily sea surface temperatures (SST; 1 km resolution) from JPL MUR SST data (2010) (https://podaac.jpl.nasa.gov/Multi-scale_Ultra-high_Resolution_MUR-SST) over a time range of 4 years (2009–2012) for each cell (see the recent proof of concept by Mangano et al.^40^ for a detailed presentation of the model and its validation).

TRO values are validated by using in situ data, collected during ad-hoc oceanographic surveys^40^. This approach assumes stationarity in biological parameters (i.e. DEB parameter values estimated for populations in one location/time are valid for populations elsewhere). We adopted the DEB parameters designed for the Mediterranean anchovy by Pethybridge et al.^50^, the degree of uncertainty of our simulations was low (see Fig. 2 in Mangano et al.^40^) and sufficiently robust to allow reliable predictions of anchovy life-history traits. We are aware that phenotypic plasticity and/or local adaptation have the potential to increase the degree of uncertainty of modeling outcomes and we suggest the use of DEB parameters values that, to the extent possible, realistically match those of local populations rather than global (species specific) parameters.

## Supplementary information

Supplementary Information
