# Supplementary material for: Seascape connectivity of European anchovy in the Central Mediterranean Sea revealed by weighted Lagrangian backtracking and bio-energetic modelling
Source: Sci Rep. 2020 Oct 29;10:18630. doi: 10.1038/s41598-020-75680-8 (PMC7596485; doi:10.1038/s41598-020-75680-8)
Supplement: Supplementary file 1 — Supplementary Information [file 41598_2020_75680_MOESM1_ESM.pdf]

Supplementary information
for
Seascape connectivity of European anchovy in the Central Mediterranean Sea revealed by weighted Lagrangian backtracking and bio-energetic modelling

Federico Falcini*[1], Raffaele Corrado[1], Marco Torri[2], Maria Cristina Mangano[3,4], Rafik Zarrad[5], Antonio Di Cintio[1], Luigi Palatella[6], Othman Jarboui[7], Hechmi Missaoui[8], Angela Cuttitta[9], Bernardo Patti[10], Rosalia Santoleri[1], Gianluca Sarà[3], Guglielmo Lacorata[1,11]

[1]*Istituto di Scienze Marine, Consiglio Nazionale delle Ricerche, UOS Roma, 00133 Rome, Italy*

[2]*Istituto per lo studio degli impatti Antropici e Sostenibilità in ambiente marino,* Consiglio Nazionale delle Ricerche, *SS Capo Granitola, 91021 Campobello di Mazara (TP), Italy*

[3]*Dipartimento di Scienze della Terra e del Mare, DiSTeM, Università degli Studi di Palermo, Edificio 16, 90128 Palermo, Italy*

[4]*Stazione zoologica Anton Dohrn, Dipartimento Ecologia Marina Integrata, Sede Interdipartimentale della Sicilia, Lungomare Cristoforo Colombo (complesso Roosevelt), 90142 Palermo, Italy*

[5]*Institut National des Sciences et Technologies de la Mer, BP. 138, 5199 Mahdia, Tunisia*

[6]*Liceo Scientifico Statale "C. De Giorgi", I-73100 Lecce, Italy*

[7]*Institut National des Sciences et Technologies de la Mer, BP 1035, 3018 Sfax, Tunisia*

[8]*Institut National des Sciences et Technologies de la Mer, 2025 Salambô, Tunisia*

[9]*Istituto di Studi sul Mediterraneo, Consiglio Nazionale delle Ricerche, SS Palermo, 90145 Palermo, Italy*

[10]*Istituto per lo studio degli impatti Antropici e Sostenibilità in ambiente marino,* Consiglio Nazionale delle Ricerche, *SS Palermo, 90149 Palermo (PA), Italy*

[11]*Center of Excellence for Telesensing of Environment and Model Prediction of Severe events (CETEMPS), Università dell'Aquila, L'Aquila, Italy*

**Corresponding author: federico.falcini@cnr.it*

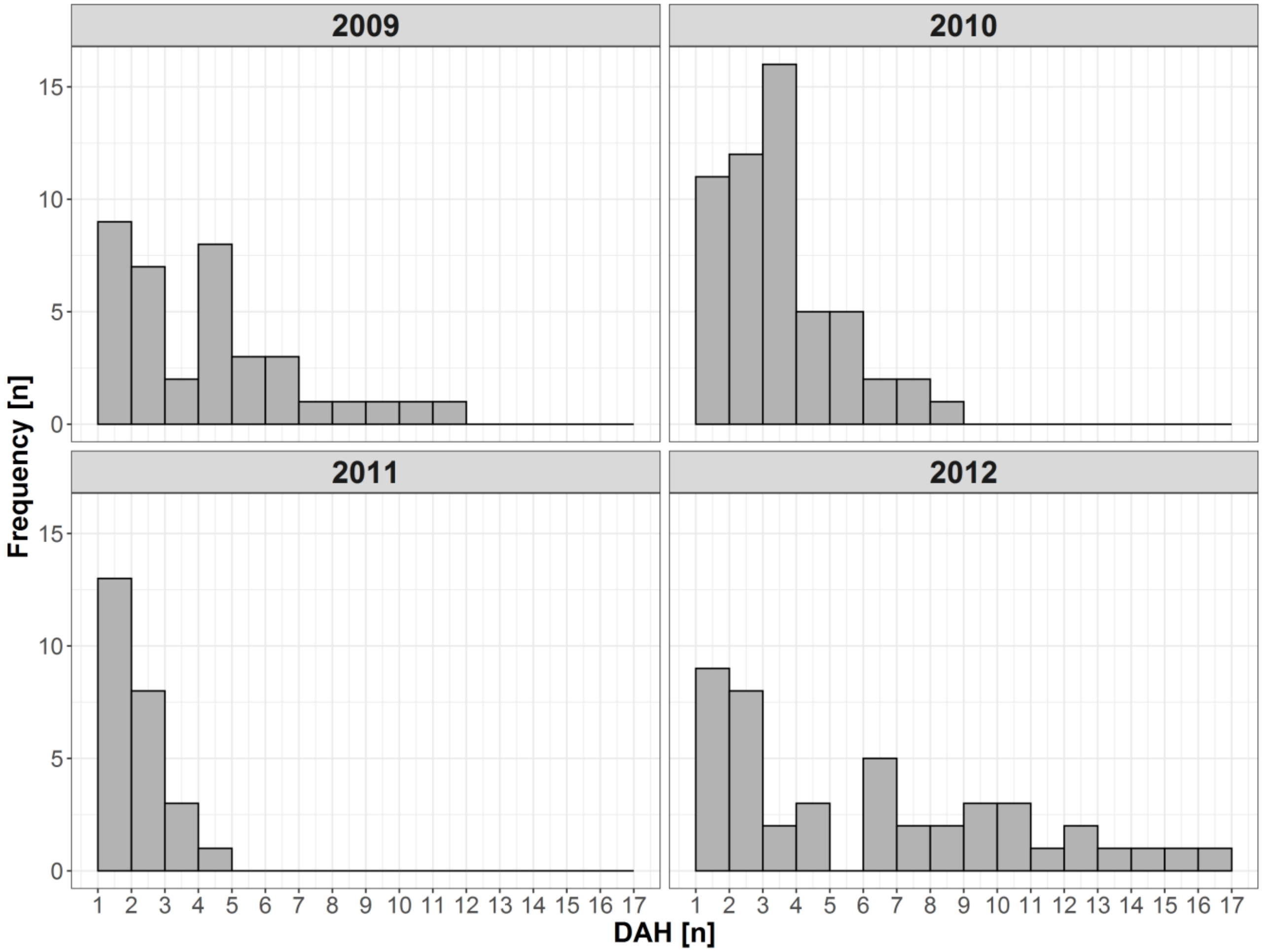

Fig. S1 – Age (days after hatching, DAH) frequency distribution estimated from the otolith analysis of European anchovy larvae collected in four surveys conducted in the Strait of Sicily (GSA16 and GSA15).

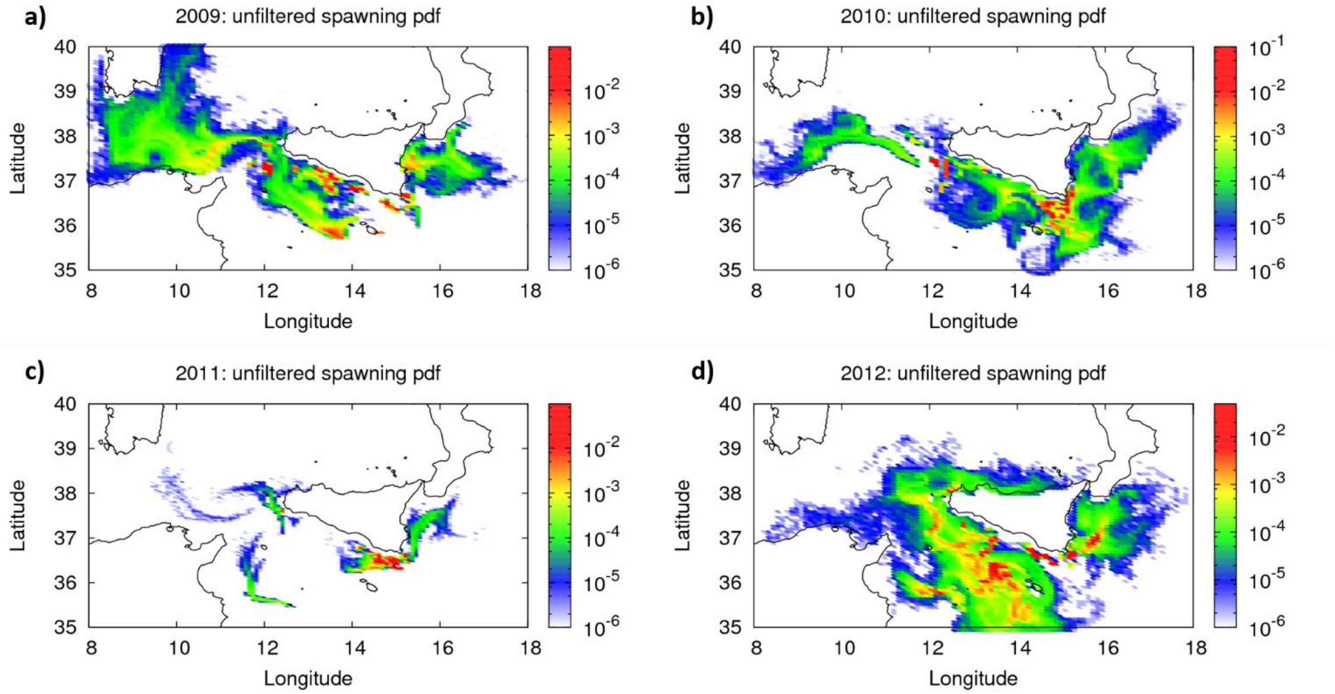

Fig. S2 – Probability distribution function (pdf) of spawning areas, as reconstructed from back-trajectories of anchovy larvae (no chlorophyll-weighted filter is applied), collected within the GSA16, in the year 2009 (a), 2010 (b), 2011 (c), and 2012 (d). Numerical back-trajectories were initialized from in-situ eggs and larvae concentration and age data (see text). Maps were generated using GIMP 2.8 (http://gimp.org).
